# Effects of a Healthy Diet on Reducing Symptoms of Premenstrual Syndrome and Improving Quality of Life among Omani Adolescents: A Randomized Controlled Open-Label Trial

**DOI:** 10.3390/ijerph20247169

**Published:** 2023-12-12

**Authors:** Maisa Hamed Al Kiyumi, Zalikha Al Belushi, Amal Al Amri, Rawan Al Musharrafi, Fathiya Al Rashdi, Sanjay Jaju, Asma Al Shidhani, Abdulaziz Al Mahrezi

**Affiliations:** 1 Department of Family Medicine and Public Health, Sultan Qaboos University Hospital, Sultan Qaboos University, Muscat 123, Oman; asmash@squ.edu.om (A.A.S.); abdulaziz@squ.edu.om (A.A.M.); 2 Department of Primary Health Care, Ministry of Health, Muscat 100, Oman; dr.almashrafi@gmail.com; 3 Ministry of Health, Muscat 100, Oman; dr98amal@gmail.com (A.A.A.); fathiya.alrashdi@moh.gov.om (F.A.R.); 4 Department of Family Medicine and Public Health, Sultan Qaboos University, Muscat 123, Oman; sanjay@squ.edu.om

**Keywords:** premenstrual syndrome, adolescent gynecology, diet, health-related quality of life, motivational follow-up, perceived stress scores (PSS)

## Abstract

Premenstrual syndrome (PMS) continues to impact the health outcomes and emotional well-being of reproductive-age women, globally. Several studies have provided conflicting evidence concerning the role of dietary approaches in improving PMS symptoms. Accordingly, this study aimed to evaluate the possible influence of a healthy diet and motivational strategies on PMS symptoms and health-related quality of life among Omani adolescents. This open-label, randomized, prospective controlled trial was conducted at two randomly selected secondary schools, in Al Seeb Willayah, in Muscat region. Adolescents with PMS symptoms, who were in grade 10 or 11, aged 16 years or above, had regular menstrual cycles, and were not known to have psychiatric disorder were included in this study. Participants in the intervention group received an individual face-to-face dietary consultation and motivational phone consultation. The health outcomes, including the PMS symptoms in both groups, and quality of life, were recorded using the Daily Record of Severity of Problems questionnaire (DRSP) and the 14-item Self-Reporting-Based Perceived Stress Scale tools, respectively. The primary outcome was the difference in the mean premenstrual symptom scores between the two groups. Secondary outcomes included the quality of life and stress levels of participants. The study period was from 1 February and ended 30 June 2021. SPSS was used to analyze the data, and intention-to-treat analysis was utilized. A total of 72 adolescents with PMS were randomized into intervention and control groups (*n* = 36 each). Both groups were similar at baseline (*p*-value > 0.05). No significant association was found between a healthy diet and PMS symptoms (*p*-value > 0.05). In addition, no significant association was found between a healthy diet and quality of life at follow-up (*p*-value = 0.216). The outcomes of this study refuted any possible relationships between a healthy diet and PMS symptoms. Accordingly, dietary consultations may not facilitate the clinical management of PMS symptoms in adolescent females.

## 1. Introduction

Reproductive-age women experience a high risk and incidence of premenstrual syndrome (PMS) and its deleterious complications [1]. It is an admixture of health complications that impact women’s physical and emotional well-being during the late luteal phase of their menstrual cycle and gradually subside with the beginning of menstruation [2]. Substantial variations in the incidence and prevalence of PMS have been reported across the globe, possibly due to differences in diagnostic approaches, including instrumentation [1]. A systematic review revealed that about 47.8% of women were affected with PMS and that the prevalence ranges from 12% in France to 98% in Iran [1,2,3,4]. Premenstrual dysphoric disorder (PMDD) diagnostic parameters were observed and met in 3–8% of reproductive-age women with PMS [5]. PMS symptoms include irritability and anger that gradually aggravate and worsen, six days before the initiation of the menstrual cycle [6]. The predominant clinical manifestations of PMS include breast tenderness, abdominal bloating, social withdrawal, depression, and anxiety [7]. The etiology of PMS is based on disturbances in the secretion and function of neurotransmitters, including gamma-aminobutyric acid (GABA) and serotonin [8,9]. The contemporary literature provides evidence substantiating the adverse impact of PMS manifestations on the daily living activities of reproductive-age women. For instance, at least one in three women with PMS were unable to independently manage their daily living activities in the absence of caretaker support [10]. In addition, data indicate the negative influence of PMS symptoms on the academic performance of university-level female students [11]. Data further indicate a strong correlation between psychiatric comorbidities and PMS manifestations; these comorbid conditions majorly include somatoform disorders, anxiety, and depression [12]. Interestingly, PMS complicates the period of puerperium and adds to the risk and incidence of depression [13]; this association suggests that the two conditions share similar features of vulnerability to changes in female gonadal hormones [12,13].

Robust evidence supports the use of selective serotonin re-uptake inhibitors (SSRIs) as a first-line treatment for PMS [14]. Data suggest that SSRIs are effective whether used during the luteal phase or continuously [14]. However, side effects are frequent, with nausea and asthenia being the most commonly described [14]. Several patient-reported surveys have revealed the non-pharmacological treatment preferences of PMS-affected women, compared to medication-based management [15]. Despite the notion of the positive health influence of dietary approaches on PMS symptoms, the current evidence does not suffice for its inclusion in treatment prescriptions in the absence of pharmacological therapy [16]. Importantly, findings from a recent study revealed an increased rate of negative affect and impaired performance with the higher consumption of carbohydrates [17]. Contrarily, evidence also indicates a statistically insignificant association between PMS symptom severity and consumption of a carbohydrate-rich diet [18]. Few studies have demonstrated a significant change in the food consumption patterns of females before menstruation and a higher risk of PMS due to an increased intake of salts, saturated fat, carbohydrates, and sugar [19,20]. Although the American College of Obstetricians and Gynecologists advises women with PMS to consume frequent small portions of complex carbohydrates and reduce the intake of sugar and salt to help with reducing symptoms of PMS, the recommendation is based on limited evidence [21]. Based on contemporary evidence, this study hypothesizes the possible beneficial role of a healthy, balanced diet in managing the clinical manifestations and the overall health and wellness of patients with PMS. This study aimed to investigate the role of face-to-face dietary recommendations and the motivational follow-up protocol in minimizing the symptoms and improving the health-related quality of life in adolescent females with PMS.

## 2. Materials and Methods

This is a prospective, open-label, randomized controlled trial of two parallel groups. It was conducted in two randomly selected secondary schools in Al Seeb Willayah, in Muscat region. The consecutive sampling approach was used to recruit the study participants. Candidates who qualified for the initial eligibility criteria were interviewed by the principal and co-principal investigators. Subjects who had one symptom or more of PMS in accordance with *The Diagnostic and Statistical Manual of Mental Disorders*, Fifth Edition (DSM5) [22], were advised to fill in the Daily Record of Severity of Problems questionnaire (DRSP). As recommended by the DSM5, women were instructed to fill in the DRSP for two consecutive months [22]. Then, those who fitted the criteria of PMS were invited to participate in our study. The study was started on 1 February 2021 and ended on 30 June 2021. The trial is registered with the WHO/Iranian Registry of Clinical Trials # IRCT20201129049526N1.

### 2.1. Inclusion and Exclusion Criteria

Adolescents who were in grade 10 or 11, aged 16 years or above, and had regular menstrual cycles were included in this study. Exclusion criteria included those who were known to have a psychiatric disorder (such as depression, generalized anxiety disorder, post-traumatic stress disorder, psychotic disorders), and diabetes. In addition, those with a history of oral contraceptive use, and those who were previously administered with, or adhered to, dietary recommendations for PMS management or those who utilized herbal remedies were further excluded. Adolescents with a confirmed diagnosis of premenstrual dysphoric disorder (PMDD) were also excluded and referred to the nearest local healthcare center for urgent management by a well-trained family physician.

### 2.2. Sample Size

The sample size for the primary outcome was calculated based on the difference in mean DRSP scores for a two-group parallel clinical trial with equal allocation. The acceptable effect at which superiority could be declared if there was a decrease in the summary DRSP score of six on the DRSP tool in the intervention group compared to the control group was used. The true difference was considered to be seven, and the conservative estimate of an expected standard deviation in the population in the trial was considered to be 1.5, resulting in an effect at a size of 0.67. For a power of 80% and α of 5%, the required sample size in each group was 28 subjects. Anticipating a dropout of 25%, the expected sample size was 35 PMS subjects in each group. nMaster 2.0 software was used to calculate the sample size [23].

### 2.3. Recruitment and Randomization

Consecutive sampling was used for the recruitment stage. Cluster randomization of the schools was carried out to minimize the contamination anticipated from recruiting subjects from the same school. Notably, the study was conducted in two randomly selected secondary schools in Al Seeb Wilayat in Muscat region. The participants in the intervention group (school A) and those in the control group (school B) were recruited from two separate schools, which were located in different areas and were maintained at an adequate distance from each other within Al Seeb Wilayat.

### 2.4. Treatment Protocol

Participants in the intervention group received individual face-to-face dietary consultations with a well-experienced clinical dietician, who evaluated the overall nutritional status of the participants, followed by an explanation of the concept of a healthy diet, including a discussion on the six food groups essential for healthy eating. The food-based dietary guidelines (FBDs), customized for Omani adolescents, were used to generate healthy, balanced diet recommendations for the participants [24]. These recommendations included carbohydrates (330–450 g), proteins (48–60 g), fiber (19–48 g), energy (2400 Kcal), calcium (600–960 mg), and salt (<5 mg/day) [24]. Subjects were specifically advised to limit extra salt, caffeine, and sugar intake. Special weighted scoops were provided to each participant in order to estimate the amounts of specific food intakes at certain meals (e.g., rice, pasta, etc.). In this study, a 24 h recall, online and self-administered, was employed to assess participants’ intake twice a week over a span of 2 months [25]. The clinical dietitian provided detailed instructions on using measuring cups and spoons to measure food intake for each food group. One technique used to estimate fiber intake involved multiplying the number of servings in each food category by the average amount of dietary fiber found in each group—1.5 g for fruits, 1.5 g for vegetables, 1.0 g for refined grains, and 2.5 g for whole grains. The dietician evaluated compliance with the dietary recommendations twice a week and offered supplementary consultations as needed. A motivational phone consultation for each participant in the intervention group was carried out by the principal and co-principal investigators once every two weeks throughout the study period. It basically included motivating the participants to comply with the dietary advice given and explore any challenges that they may have encountered that might impede their adherence. Moreover, one parent was instructed by phone every two weeks to ensure the compliance of his/her daughter to dietary advice. Subjects in the control group did not receive any dietary advice at baseline. However, dietary counselling for each subject in the control group was provided at the end of the study by the same dietician. Similar recommendations regarding physical exercise were provided to both the intervention and control groups at baseline.

### 2.5. Assessment Approach

At baseline, the sociodemographic questionnaire was administered to both study groups, which included basic sociodemographic features such as age, and chronic conditions such as thyroid disease, medication use, smoking, alcohol status and substance use. The dietitian evaluated the dietary habits of participants in both the control and intervention groups. The assessment covered the number of servings of milk and dairy products, meat, fish, legumes, vegetables, fruits, refined carbohydrate-rich foods, caffeine, and carbonated drinks, as well as fat-rich foods. In addition, at baseline, and by the end of the study, the DRSP (Arabic version) was disseminated to participants of the control and intervention groups. The DRSP tool is known for its high sensitivity, specificity, and validity in calculating PMS and PMDD incidence rates [26]. Moreover, it is a highly reliable instrument based on its capacity to track PMS symptoms and their alterations during menstruation. It is also known to evaluate treatment responses in PMS-affected patients [26]. A 24-item DRSP tool collects data regarding overall impairment as well as physical and emotional symptoms. Each DRSP item needs to be rated by the participants per day and is guided by a six-point scale to determine the symptom severity. DRSP also collects data concerning the dates and duration of menstrual bleeding [26]. PMDD is diagnosed if the subject meets all the following criteria: (i) scores at least four in one of more symptoms of depression (items 2–4), anxiety (item 5), lability (item 6), and anger (items 7 and 8) for two days or more before menses; (ii) scores at least four in at least five of the symptoms listed in items 9–22 for two days or more before menses; (iii) scores at least four in at least one of the three impairment items (items 23–25). If the subject does not meet one or more of the above criteria, then she will be diagnosed as having PMS. Neither PMS nor PMDD will be diagnosed if the subject denies any symptoms. The Arabic version of DRSP has been validated and is considered to be a sensitive and reliable tool [11]. Notably, as women were diagnosed as having PMS based on their response to DRSP over a two-month period, the second month’s response was considered as the baseline response. The difference in the mean value of DRSP during the one week before menses between the two groups at the end of the study was considered as a primary outcome for this study.

Moreover, a PSS was filled out by the participants at baseline and by the end of the study. It is a self-reporting instrument that assesses the degree to which the individual perceives his/her situation as stressful [27]. It consists of 14 items related to the thoughts and feelings a person had during the previous month. The Arabic version has already been validated [28] and the differences in the mean score between the two groups at the end of the study was considered as the secondary outcome of this study. Supplementary Figure S1 presents the screening and the randomization processes, while Supplementary Figure S2 illustrates the study procedures and equipment.

### 2.6. Statistical Analysis

The trial was reported using the intention-to-treat analysis method. The difference in the DRSP scores (primary outcome) and PSS scores (secondary outcomes) from baseline to the end of the intervention was compared between the randomized groups using analysis of covariance (ANCOVA), and differences in scores reported as adjusted mean differences and 95% confidence intervals (i.e., adjusted for the baseline score as the covariate). Categorical outcomes were compared between groups using Chi-square tests. All tests were two-tailed and a *p* value of less than 0.05 was considered statistically significant.

## 3. Results

**Figure 1:**
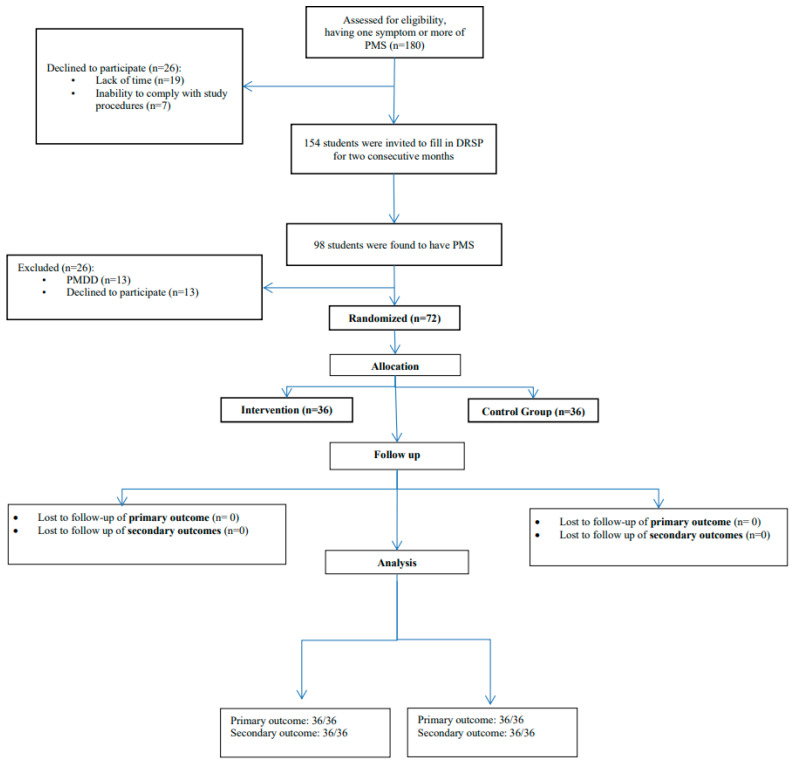
Flow chart.

The screening process was initiated after the initial interviews of candidates by the principal and co-principal investigators (Figure 1).

Ninety participants from each school were notified and invited to register and participate in the screening process (*n* = 180). However, 26 students (12 from school A and 14 from school B) did not provide their consent for the screening process due to time constraints and unwillingness to adhere to the study protocols. These were subsequently excluded. In addition, PMS was confirmed in 48 school A and 50 school B students, following their DRSP inputs for two consecutive months. Six students from school A and seven from school B were diagnosed with PMDD and were referred to the nearest local health center. Finally, 42 and 43 students from school A and school B, respectively, were found to be eligible to participate in this randomized controlled study. Furthermore, six students from school A and seven from school B could not participate in the study due to a time deficit. Eventually, 36 students from school A and an equal number from school B were categorized into the control and intervention groups, respectively. No loss to follow-up was reported within the study tenure.

Table 1 presents the baseline attributes of both the intervention and control groups (*n* = 36, each); their comparative analysis revealed no statistically significant differences (all *p* > 0.05). Additionally, no statistically significant differences between the study groups were reported for menstrual cycle length (*p* = 0.493), bleeding amount (*p* = 0.326), dysmenorrhea (*p* = 0.54), and absenteeism from school (*p* = 0.430). No statistically significant differences between the study groups were reported for the dietary intake variables, including fish, legumes, vegetables, fruits, refined carbohydrate-rich diet, sugar, caffeine, carbonated drinks, chocolate and related food items, and fat-rich diets (all *p* > 0.05). Differences in other variables, including mother’s education, father’s education, and exercise also lacked statistical significance (all *p* > 0.5).

It was evident that, prior to the intervention, a majority of participants did not follow healthy eating patterns. A significant number showed inadequate fiber intake, as reflected in their limited consumption of fruits, vegetables, and whole-grain food items. Furthermore, there was a noticeable deficiency in their daily water intake. Table 2 provides an overview of the food intake of participants in the intervention group during the pre-intervention period, and at weeks 1, 4, and 8 in the post-intervention period, as assessed using the 24 h recall instrument.

The analysis of covariance (ANCOVA) of subsections (depressive symptoms; physical symptoms and anger and irritability) and also the total scores of the Daily Record of Severity of Problems questionnaire (DRSP) in the two groups (Table 2) revealed no significant differences at the end of the first and second month follow-ups, as compared to the baseline scores (all *p* > 0.05) (Table 3).

With regards to the PSS outcomes at baseline and after two months between the control and intervention groups, the findings were also devoid of any statistically significant change (*p* = 0.216) (Table 4).

## 4. Discussion

Our study revealed no significant association between healthy and well-balanced dietary intake and symptoms of premenstrual syndrome based on the daily record of severity of problems questionnaire (DRSP). Additionally, no significant association was found between a healthy diet in adolescents with premenstrual syndrome, and quality of life as measured by the PSS.

Our results support the outcomes of several studies that demonstrate an insignificant relationship between PMS symptoms and carbohydrate intake [18,29]. Furthermore, evidence refuted any significant association between PMS and fat intake, including trans/polyunsaturated/monounsaturated fats [30]. However, findings from some studies have highlighted the possible role of saturated fat in minimizing PMS predisposition in women [30,31]. Outcomes from some other studies further negate any significant relationship between PMS risk and total protein/amino acid intake in females [32]. Few studies demonstrate an inverse relationship between PMS symptoms and high coffee consumption or increased carbohydrate intake [33,34].

Findings from a UAE-based study by Hashim et al. highlighted the detrimental effects of salt-based diets, high sugar intake, fat-based food intake, high-calorie diets, and smoking on physical symptoms of PMS [19]. Additionally, they revealed a statistically significant inverse relationship between PMS-related behavioral symptoms and fruit consumption. Another Korea-based cross-sectional study revealed no significant relationship between PMS risk and the consumption of alcohol, meat, or traditional diets [35]. Similarly, a recent cross-sectional study revealed a significant association between severe premenstrual syndrome and excess salt intake among high school students in Iran [36]. A recent review paper by Simini and Turcanu provided substantial evidence indicating no statistically significant association between PMS and the intake of macronutrients, including fiber, carbohydrates, fat, and proteins [31]. Results from the same study also indicated the impact of micronutrients on PMS symptoms; these micronutrients include herbal supplements, vitamin B complex, vitamin D, magnesium, and calcium [31]. Another qualitative study by Quaglia et al. indicated a strong association between PMS symptom burden and total energy intake, based on food items, including micro/macronutrients [37]. Overall, most studies in the literature support our findings concerning the absence of a statistically significant relationship between PMS symptoms and a healthy, balanced diet.

The pathology of PMS and the diversity of its symptoms appear to impact these results, which require further evaluation via prospective studies. To date, no clinical study can explain and elaborate on the multifactorial symptomatology of PMS [1]. Scant evidence is available on the possible impacts of psychosocial complications and hormonal imbalances on PMS symptoms, including their severity, risk, and incidence. The pathophysiology of PMS is based on the activity of catecholamine, serotonin, opioids, GABA, and progesterone [38]. Importantly, increased progesterone sensitivity with a preexisting serotonin deficiency is also considered responsible for PMS symptom severity [38]. Evidence suggests that certain dietary factors, such as tryptophan, which is present in chicken, fish, eggs, nuts, and dairy products, serve as a precursor to serotonin synthesis [39]. In addition, certain vitamins, and minerals, such as vitamin B6, vitamin B12, folate, and magnesium, are involved in the conversion of tryptophan to serotonin [40]. Other potential PMS-triggering factors include genetic factors, electrolyte deficiency, insulin resistance, improper hypothalamic–pituitary–adrenal axis activity, abnormal glucose metabolism, and elevated prolactin. The sympathetic activity, amplified by stress levels, potentiates uterine contraction, which eventually increases the severity and frequency of menstrual pain [38]. Several comorbid conditions, including hyperprolactinemia and Cushing syndrome, disturb the normal levels of cortisol, thyroid stimulating hormone, estradiol, and follicular stimulating hormone in women, thereby predisposing them to PMS [41].

### 4.1. Limitations

This study has some limitations. First, the small sample size impacts the generalizability of the findings. In addition, the lack of blinding, despite the study’s randomized controlled design, increased the risk of selection bias. Moreover, the use of self-reported questionnaires might have further impacted the reliability of results. Lastly, the lack of an objective scale with which to measure adherence to dietary advice might have had a negative impact on the final results.

### 4.2. Recommendations

In spite of a variety of cross-sectional studies evaluating the relationships between PMS and dietary patterns, the paucity of evidence to date has substantiated the need for further prospective randomized studies with larger sample sizes to understand the potential factors impacting the PMS manifestations. These randomized studies should also explore the possible role of geographical variations and culture-based diets on PMS progression and its clinical manifestations in adolescent and adult females. Clinical trials should further explore the interactions between diets and medications and their possible influence on PMS development in females of various age groups.

## 5. Conclusions

The overall findings from this study revealed a nonsignificant association between healthy balanced diet, motivational follow-ups, and PMS symptom improvements. Although our research did not find statistically significant results supporting the effectiveness of dietary modifications and motivational support as treatments for PMS, it is crucial to recognize the study’s limitations, including the small sample size. Future prospective studies with larger and more diverse sample sizes are needed to allow for a more robust analysis of the relationships between dietary habits, motivational interventions, and PMS symptomatology.

## Figures and Tables

**Table 1:** Baseline characteristics.

Variables			Control (*n* = 36)	Intervention (*n* = 36)	*p*-Value
Age, mean (SD)			15.92 (0.44)	15.97 (0.38)	0.566
BMI, mean (SD)			20.80 (3.22)	21.17 (3.71)	0.661
Age at menarche, mean (SD)			12.56 (0.77)	12.26 (1.36)	0.257
Days of bleeding during menses, *n* (%)		<3 days	1 (2.8%)	0 (0%)	0.602
		3–7 days	30 (83.3%)	31 (86.1%)	
		>7 days	5 (13.9%)	5 (13.9%)	
Length of menstrual cycle, *n* (%)		<21 days	4 (11.1%)	6 (16.7%)	0.493
		21–35 days	31 (86.1%)	30 (83.3%)	
		>35 days	1 (2.8%)	0 (0%)	
Amount of bleeding (changing pads every 1–2 h or passage of clots >2.5 cm), *n* (%)		Yes	15 (41.7%)	11 (30.6%)	0.326
		No	21 (58.3%)	25 (69.4%)	
Dysmenorrhea, *n* (%)		Yes	32 (88.9%)	31 (86.1%)	0.54
		No	4 (11.1%)	5 (13.9%)	
Absence from school, *n* (%)		Yes	8 (22.2%)	12 (33.3%)	0.293
		No	28 (77.8%)	24 (66.7%)	
Family size			5.09 (1.98)	5.65 (3.08)	0.377
Mother’s educational level		University	15 (41.7%)	7 (19.4%)	0.197
		Secondary school	15 (41.7%)	18 (50%)	
		Primary school	3 (8.3%)	6 (16.7%)	
		Illiterate	3 (8.3%)	5 (13.9%)	
Father’s educational level		University	19 (52.8%)	11 (30.6%)	0.177
		Secondary school	13 (36.1%)	18 (50%)	
		Primary school	3 (8.3%)	6 (16.7%)	
		Illiterate	0 (0%)	1 (2.8%)	
Chronic diseases (thyroid, hypertention, heart disease, kidney disease), *n* (%)		Yes	0 (0%)	0 (0%)	NA
		No	36 (100%)	36 (100%)	
Exercise, *n* (%)		Yes	25 (69.4%)	29 (80.6%)	0.276
		No	11 (30.6%)	7 (19.4%)	
Dietary Habits	Milk and dairy products, *n* (%)	No	1 (2.8%)	1 (2.8%)	0.602
		1 time per month	0 (0%)	3 (8.3%)	
		2 times per month	2 (5.6%)	2 (5.6%)	
		1–2 times per week	13 (36.1%)	11 (30.6%)	
		Once daily	12 (33.3%)	9 (25%)	
		More than one time per day	8 (22.2%)	9 (25%)	
	Meat, *n* (%)	No	3 (8.3%)	1 (2.8%)	0.267
		1 time per month	3 (8.3%)	1 (2.8%)	
		2 times per month	6 (16.7%)	7 (19.4%)	
		1–2 times per week	17 (47.2%)	18 (50%)	
		Once daily	4 (11.1%)	8 (22.2%)	
		More than one time per day	3 (8.3%)	0 (0%)	
	Fish, *n* (%)	No	9 (25%)	8 (22.2%)	0.843
		1 time per month	1 (2.8%)	2 (5.6%)	
		2 times per month	4 (11.1%)	2 (5.6%)	
		1–2 times per week	20 (55.6%)	20 (55.6%)	
		Once daily	2 (5.6%)	2 (5.6%)	
		More than one time per day	0 (0%)	1 (2.8%)	
	Legumes, *n* (%)	No	3 (8.3%)	9 (25%)	0.073
		1 time per month	6 (16.7%)	3 (8.3%)	
		2 times per month	2 (5.6%)	7 (19.4%)	
		1–2 times per week	16 (44.4%)	13 (36.1%)	
		Once daily	6 (16.7%)	2 (5.6%)	
		More than one time per day	3 (8.3%)	1 (2.8%)	
	Vegetables, *n* (%)	No	0 (0%)	5 (13.9%)	0.086
		1 time per month	3 (8.3%)	1 (2.8%)	
		2 times per month	2 (5.6%)	4 (11.1%)	
		1–2 times per week	4 (11.1%)	5 (13.9%)	
		Once daily	20 (55.6%)	11 (30.6%)	
		More than one time per day	7 (19.4%)	9 (25%)	
	Fruits, *n* (%)	No	2 (5.6%)	1 (2.8%)	0.735
		1 time per month	1 (2.8%)	3 (8.3%)	
		2 times per month	2 (5.6%)	3 (8.3%)	
		1–2 times per week	7 (19.4%)	9 (25%)	
		Once daily	15 (41.7%)	10 (27.8%)	
		More than one time per day	9 (25%)	9 (25%)	
	Refined Carbohydrate rich food, *n* (%)	No	0 (0%)	2 (5.6%)	0.716
		1 time per month	1 (2.8%)	2 (5.6%)	
		2 times per month	3 (8.3%)	4 (11.1%)	
		1–2 times per week	13 (36.1%)	11 (30.6%)	
		Once daily	14 (38.9%)	12 (33.3%)	
		More than one time per day	5 (13.9%)	4 (11.1%)	
	Sugar, caffeine and carbonated drinks, *n* (%)	No	1 (2.8%)	2 (5.6%)	0.702
		1 time per month	1 (2.8%)	4 (11.1%)	
		2 times per month	4 (11.1%)	3 (8.3%)	
		1–2 times per week	9 (25%)	8 (22.2%)	
		Once daily	12 (33.3%)	9 (25%)	
		More than one time per day	9 (25%)	8 (22.2%)	
	Chocolate and food containing chocolate, *n* (%)	No	0 (0%)	1 (2.8%)	0.230
		1 time per month	0 (0%)	1 (2.8%)	
		2 times per month	0 (0%)	4 (11.1%)	
		1–2 times per week	10 (27.8%)	9 (25%)	
		Once daily	16 (44.4%)	13 (36.1%)	
		More than one time per day	10 (27.8%)	7 (19.4%)	
	Fat rich food, *n* (%)	No	0 (0%)	2 (5.6%)	0.340
		1 time per month	0 (0%)	1 (2.8%)	
		2 times per month	1 (2.8%)	3 (8.3%)	
		1–2 times per week	14 (38.9%)	16 (44.4%)	
		Once daily	10 (27.8%)	6 (16.7%)	
		More than one time per day	10 (27.8%)	7 (19.4%)	
DRSP		Depressive symptoms @baseline	79.06 (29.77)	79.62 (26.61)	0.932
		Physical symptoms @baseline	50.94 (17.96)	49.86 (17.79)	0.797
		Anger and Irritability @baseline	29.67 (12.53)	27.78 (11.05)	0.498
		Total @baseline	326.86 (111.10)	321.08 (100.35)	0.816
PSS		PSS at baseline	28.03 (7.69)	27.44 (7.81)	0.750

**Table 2:** 24 h recall analysis of participants in the intervention group at pre-intervention period, and at weeks 1, 4, and 8 at post-intervention period.

Food Item Number	Dietary Components/ Food Groups	Recommended Servings/ Amounts per Day	Pre-Intervention (*n* = 36)		Post-Intervention (*n* = 36)					
					Week 1 (*n* = 36)		Week 4		Week 8	
			Less Than Recommendations, *n* (%)	Within Recommendations, *n* (%)	Less Than Recommendations, *n* (%)	Within Recommendations, *n* (%)	Less Than Recommendations, *n* (%)	Within Recommendations, *n* (%)	Less Than Recommendations, *n* (%)	Within Recommendations, *n* (%)
1	Fruits	2- 4 servings	30 (83.3%)	6 (16.7%)	24 (66.7%)	12 (33.3%)	8 (22.2%)	28 (77.8%)	4 (11.1%)	32 (88.9%)
2	Vegetables	3–5 servings	30 (83.3%)	6 (16.7%)	28 (77.8%)	8 (22.2%)	9 (25.0%)	27 (75.0%)	3 (8.3%)	33 (91.7%)
3	Milk & Dairy products	2–3 servings	25 (69.4%)	11 (30.6%)	21 (58.3%)	15 (41.7%)	10 (27.8%)	26 (72.2%)	4 (11.1%)	32 (88.9%)
4	Meat, Chicken & Poultry	4–5 oz (120–150 g)	11 (30.6)	25 (69.4%)	10 (27.8%)	26 (72.2%)	6 (16.7%)	30 (83.3%)	6 (16.7%)	30 (83.3%)
5	Legumes	35–70 g	29 (80.6%)	7 (19.4%)	26 (72.2%)	10 (27.8%)	9 (25.0%)	27 (75.0%)	4 (11.1%)	32 (88.9%)
6	Bread, rice, pasta & their substitutes	180–240 g (4–7 servings)	10 (27.8%)	26 (72.2%)	7 (19.4%)	29 (80.6%)	4 (11.1%)	32 (88.9%)	2 (5.6%)	34 (94.4%)
7	Nuts & Seeds	15- 20 g	22 (61.1%)	14 (38.9%)	16 (44.4%)	20 (55.6%)	10 (27.8%)	26 (72.2%)	3 (8.3%)	33 (91.7%)
8	Fiber	25–35 g	31 (86.1%)	5 (13.9%)	27 (75.0%)	9 (25.0%)	9 (25.0%)	27 (75.0%)	4 (11.1%)	32 (88.9%)
9	Water	8 cups (240 mL each cup) per day-2 L	29 (80.6%)	7 (19.4%)	24 (66.7%)	12 (33.3%)	8 (22.2%)	28 (77.8%)	3 (8.3%)	33 (91.7%)
**Food Item Number**	**Dietary Components/** **Food Groups**	**Recommended Servings/** **Amounts per Day**	**Higher Than ** **Recommendations, *n* (%)**	**Within ** **Recommendations, *n* (%)**	**Higher Than** **Recommendations, *n* (%)**	**Within ** **Recommendations, *n* (%)**	**Higher Than ** **Recommendations, *n* (%)**	**Within ** **Recommendations, *n* (%)**	**Higher Than ** **Recommendations, *n* (%)**	**Within ** **Recommendations, *n* (%)**
10	Fats/oils	25–30 mL/g	30 (83.3%)	6 (16.7%)	27 (75.0%)	9 (25.0%)	6 (16.7%)	30 (83.3%)	4 (11.1%)	32 (88.9%)
11	Sugar and Sugary drinks and sweets	<10% of the total calories	28 (77.8%)	8 (22.2%)	27 (75.0%)	9 (25.0%)	8 (22.2%)	28 (77.8%)	3 (8.3%)	33 (91.7%)
12	Salt/Sodium	<5 g of salt or 2.3 g of sodium (2300 mg/day)	29 (80.6%)	7 (19.4%)	28 (77.8%)	8 (22.2%)	9 (25.0%)	27 (75.0%)	2 (5.6%)	34 (94.4%)

**Table 3:** Analysis of Covariance (ANCOVA) of subsections and total scores of the Daily Record of Severity of Problems questionnaire (DRSP).

	Control Mean (SD)	Intervention Mean (SD)	*p*-Value
Depressive symptoms @baseline	79.06 (29.77)	79.62 (26.61)	
Depressive symptoms @1 month	73.58 (4.05)	71.68 (4.17)	0.744 *
Depressive symptoms @2 months	74.24 (3.68)	65.59 (3.75)	0.103 **
Physical symptoms @baseline	50.94 (17.96)	49.86 (17.79)	
Physical symptoms @1 month	46.43 (2.81)	49.42 (2.90)	0.462 *
Physical symptoms @2 months	47.46 (2.72)	45.90 (2.80)	0.691 **
Anger and Irritability @baseline	29.67 (12.53)	27.78 (11.05)	
Anger and Irritability @1 month	26.27 (1.54)	25.78 (1.59)	0.825 *
Anger and Irritability @2 months	26.16 (1.45)	23.56 (1.50)	0.218 **
Total @baseline	326.86 (111.10)	321.08 (100.35)	
Total @1 month	295.32 (14.99)	292.36 (15.42)	0.891 *
Total @2 months	298.23 (13.26)	268.55 (13.64)	0.124 **

* Change from baseline to end of 1 month. ** Change from baseline to end of 2 months.

**Table 4:** Analysis of covariance (ANCOVA) of perceived stress scores (PSS).

	Control Mean (SD)	Intervention Mean (SD)	*p*-Value
PSS @baseline	28.03 (7.69)	27.44 (7.81)	
PSS @2 months	26.42 (0.671)	27.61 (0.67)	0.216 **

** Change from baseline to end of 2 months.

## Data Availability

Data supporting the reported results can be provided by the corresponding authors upon request.

## Supplementary Materials

The following supporting information can be downloaded at: https://www.mdpi.com/article/10.3390/ijerph20247169/s1, Figure S1: Screening and Randomization. Figure S2: Study Procedures and Equipment.
