# Supplementary material for: Effects of a Healthy Diet on Reducing Symptoms of Premenstrual Syndrome and Improving Quality of Life among Omani Adolescents: A Randomized Controlled Open-Label Trial
Source: Int J Environ Res Public Health. 2023 Dec 12;20(24):7169. doi: 10.3390/ijerph20247169 (PMC10742710; doi:10.3390/ijerph20247169)
Supplement: Supplementary file 1 [file ijerph-20-07169-s001.zip › ijerph-2706598-supplementary.pdf]

Supplementary Materials

Supplementary Figures:

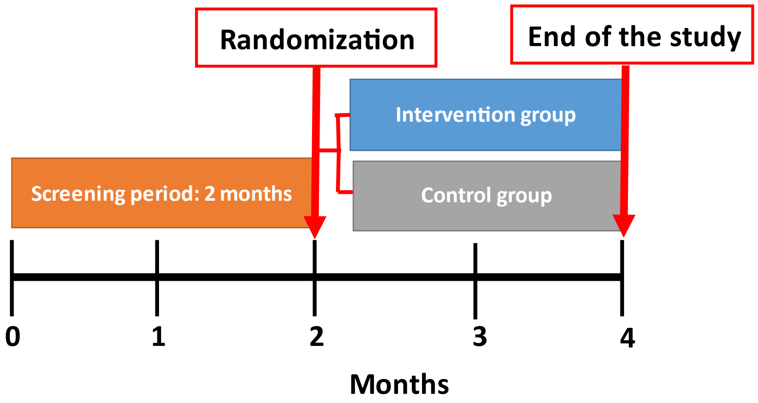

Figure S1. Screening and randomization.

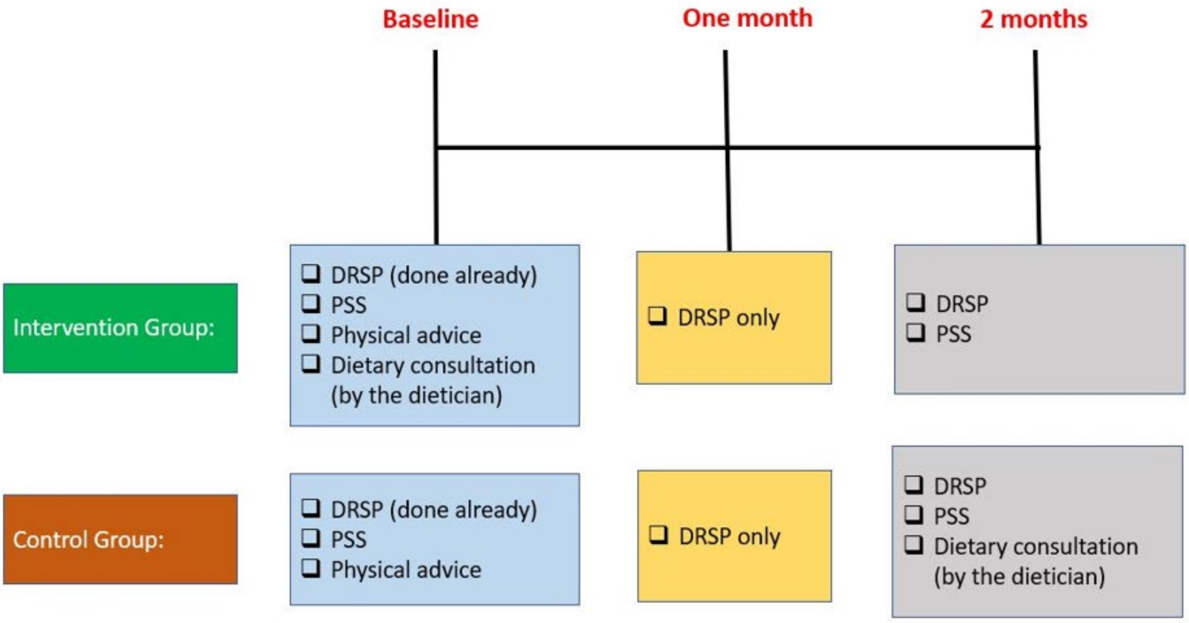

Figure S2. Study procedures and equipment.
